# Whole body vibration ameliorates anxiety-like behavior and memory functions in 30 months old senescent male rats

**DOI:** 10.1016/j.heliyon.2024.e26608

**Published:** 2024-02-16

**Authors:** Tamás Oroszi, Klára Felszeghy, Paul G.M. Luiten, Regien G. Schoemaker, Eddy A. van der Zee, Csaba Nyakas

**Affiliations:** a Department of Molecular Neurobiology, Groningen Institute for Evolutionary Life Sciences (GELIFES), University of Groningen, Groningen, the Netherlands; b Research Center for Molecular Exercise Science, Hungarian University of Sports Science, Budapest, Hungary; c Department of Morphology and Physiology, Health Science Faculty, Semmelweis University, Budapest, Hungary

**Keywords:** Passive exercise, Advanced aging, Open field activities, Spatial and novel object recognition

## Abstract

Whole body vibration (WBV) is a form of passive exercise that offers an alternative physical training to aged individuals with limitations in their physical and mental capabilities. The aim of the present study was to explore the therapeutic potential of five weeks of WBV on anxiety-like behaviors as well as learning and memory abilities in senescent thirty months old rats. Animals were exposed to 5 min vibration twice per day, five times per week during the five consecutive weeks. Pseudo WBV treated animals served as controls. After five weeks of WBV treatment, animals were tested for anxiety-like behavior by the open field test and for spatial and object memory functions by the novel and spatial object recognition tests, respectively. As a result, anxiety-like and exploratory behaviors were significantly improved in the WBV treated group compared to the pseudo WBV group. Furthermore, WBV treatment increased discrimination performance in both spatial and object memory function testing. These results indicate that WBV treatment in thirty months old rats seems to have comparable beneficial effects on age-related emotional and cognitive performance as what has been reported in younger age groups.

## Highlights

• WBV increases novelty-induced locomotor activity of 30 months old rats in open field.
• WBV attenuates anxiety-like behaviors of the 30 months old senescent rats.
• WBV ameliorates both spatial and object memory dysfunctions of the aged rats.
• WBV may be a treatment strategy in brain disorders related to senescence.

## 1 Introduction

Advanced aging poses one of the most relevant health and social challenges for today's society. The aging process is a multifactorial and partly irreversible process that leads to functional and structural deteriorations in various physiological systems. In the last decade, ample reviews have outlined the most prominent age-related changes in the context of skeletal muscle [1], bone [2], vasculature [3], cardiovascular [4] and skin systems [5]. Additionally, aging can have detrimental effects on the central nervous system (CNS), including the neural and sensory-motor systems. Notably, extensive reviews have demonstrated that aging in the CNS is accompanied by altered mitochondrial physiology [6], impaired structural and functional brain connectivity [7], as well as by declines in cognitive capabilities [8] and neurogenesis [9]. These faculties highlight the intricate complexity of the aging process. Overall, the most critical facet of aging is to diminish the living system's capacity, including the brain, to remodel, regenerate and repair its own homeostasis [10,11]. Furthermore, as aging progresses, these processes can become increasingly compromised [10,12].

Significant efforts have been devoted to preclinical investigations focusing on cognitive impairments and potential therapeutic interventions to maintain good physical and mental well-being during advanced aging. For instance: it has been established that aged rodents show impairments in their learning and memory functions, as observed in tasks such as the Morris Water Maze task [13,14], the spatial object recognition (SOR) and novel object recognition (NOR) [15,16]. Additionally, they often display elevated levels of anxiety, depression and/or hyperemotionality-like behavioral patterns, as evidenced by tests such as the open field (OFT), elevated-T maze, or plus maze [[17], [18], [19]]. Currently, extensive reviews have summarized the impact of pharmacological [[20], [21], [22]] and nutritional interventions [23,24] on memory functions and behavior. Furthermore, numerous preclinical studies involving rodents suggest that different types of active exercise interventions can beneficially modulate memory functions and anxiety-like behavior [[25], [26], [27], [28], [29]]. These different modalities of active exercise are commonly studied in the preclinical research and are also prevalent in human practice [30,31]. However, their applicability and efficiency for aged populations are often constrained due to increased physiological and mental limitations they may experience [32].

Whole body vibration (WBV), a type of passive exercise, has emerged as a potential complementary approach to improve physical and mental capabilities. Over the last decades, several studies have described the beneficial effects of WBV on musculoskeletal and neuromuscular functions in both humans [33,34] and rodents [35]. Besides its impact on muscular functions, behavioral and cognitive effects of WBV have also garnered some attention recently. Although previous studies have shown considerable variability in WBV parameters such as frequency (in Hz), amplitude (in mm), the duration of exposure (in min) and covered a wide spectrum in the characteristics of the subjects, WBV seems to be a feasible strategy for stimulating the central nervous system [[36], [37], [38], [39], [40]]. Regarding novelty-induced behavior, some studies have demonstrated that WBV improves unprompted locomotor activity, as well as learning and memory processes in rodents [41,42]. Furthermore, our research group recently showed that WBV improves anxiety-like behavior and spatial memory in 18 months old Wistar rats [43,44]. Several studies have also described the therapeutic potential of WBV interventions on brain functions in different stroke/injury models [42,[45], [46], [47], [48]], Parkinson's [39,49] and Alzheimer's diseases [50,51], chronic restraint stress [41], morphine withdrawal [52], and post-operative recovery [53].

Preclinical studies have demonstrated that active exercise can improve anxiety and memory functions in 24 months old rats [28,29]. Other studies have shown that voluntary and involuntary active exercise can contribute to enzymatic glycolytic alterations of type 2 B skeletal muscle fibers, as well as prevention of vascular dysfunctions, mitochondrial stress, and inflammation in 27–30 months old rodents [54,55]. In our previous experiments, we observed significant improvements in anxiety-like behavior and spatial memory in 18 months old rats following WBV stimulation [43,44]. However, it remains to be revealed whether these effects of WBV can still be induced in very old, senescent rats (30 months of age). For the present study, based on our previous findings, we aimed to investigate how WBV exposure influences anxiety-like behavior and memory functions in senescent (30 months) rats highlighting the potential benefits of passive exercise in advanced age.

## 2 Materials and methods

### 2.1 Animals

Twenty-four male Wistar rats were used in this study. Male animals were chosen based on our previous study, in which, 18 months old male animals expressed higher level of anxiety-like behavior and responsiveness to WBV exposure compared to the female animals [43]. Animals were bred and raised in our own animal facility under laboratory conditions, with a 12–12 dark/light cycles (light on at 7:00 a.m.), with constant temperature of 22 ± 2 °C, along with humidity level of 50 ± 10%. They were pair housed (2 animals per cage) and had ad libitum access to food and water. All procedures were approved by the animal ethical committee of the Hungarian University of Sports Science (TE-KEB/No3/2020) and animals were used in accordance with the guidelines of the European Union Council Directive (86/609/European Economic Community). The health status and body weight of the animals were monitored on a weekly basis throughout the experimental period.

### 2.2 Experimental procedures

**Fig. 1:**
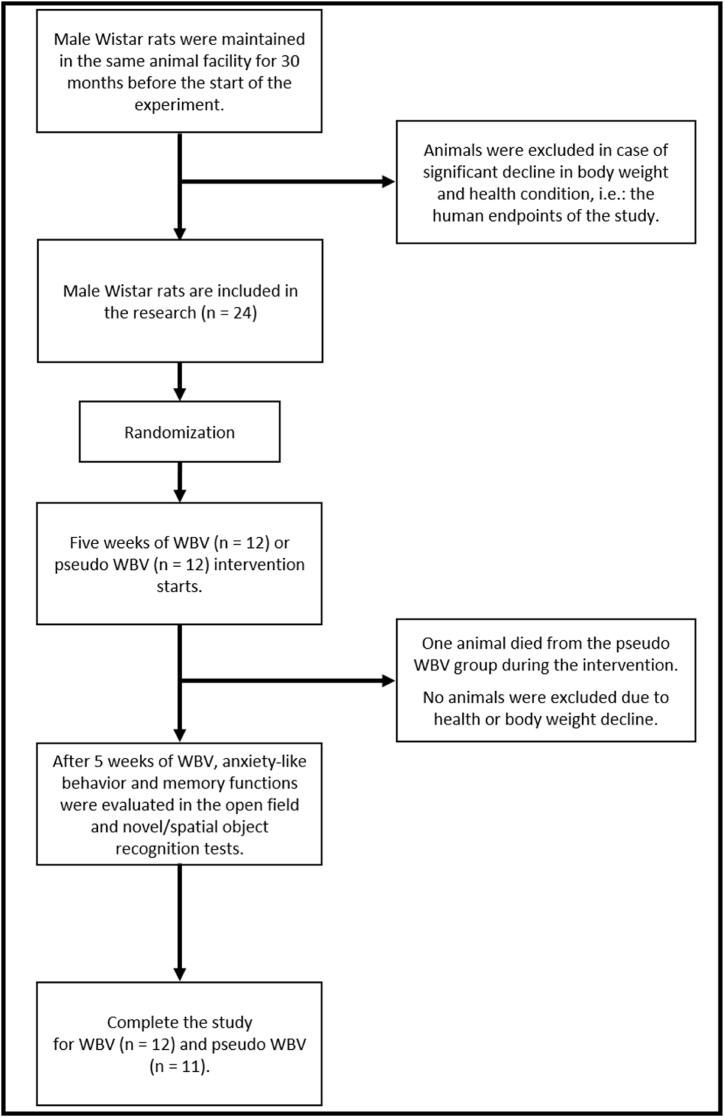
**Experimental flowchart diagram.** Male Wistar rats were maintained in the animal facility for 30 months before the Whole Body Vibration (WBV) or pseudo WBV intervention. Animals started the WBV intervention at the age of 30 months and were tested for anxiety-like behavior and memory functions. Main selection and exlusion criterias are mentioned at each steps of the study.

**Fig. 2:**
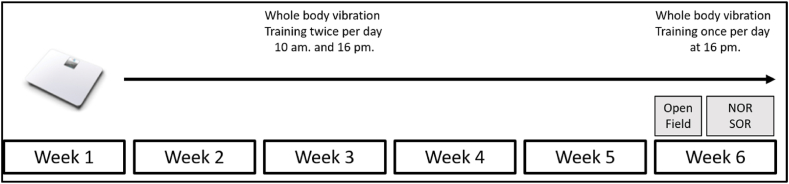
**Experimental design.** Thirty months old male Wistar rats underwent a long-term (5 weeks) Whole Body Vibration (WBV) or pseudo WBV intervention involved twice daily exposure of 5 min. After 5 weeks, only morning test procedures were conducted to evaluate anxiety-like behavior and memory functions. The used Marodyne plate is depicted on the left side.

Male animals at the age of 30 months were randomly allocated to either the WBV (n = 12) or a pseudo WBV (n = 12) treated group (see Fig. 1). Following a five-week period of WBV or pseudo WBV treatment, animals were tested for spatial and object aspects of memory functions (NOR and SOR tasks) and anxiety-like behavior (OFT). All treatments and testing procedures were conducted in a separate test room with the same environmental conditions as the housing room. One animal from the pseudo WBV group died during the intervention period. The experimental design is illustrated in Fig. 2.

### 2.3 Whole body vibration procedure

In this study, we followed the new reporting guidelines for WBV studies in animals [56]. Animals underwent two sessions of 5 min WBV per day (with one session at 10 a.m. and another at 16 p.m.), five times per week, for a total of 5 consecutive weeks. Six hours long time gap was chosen between the two daily sessions because it is assumed that both the rapidly and slowly adapting skin mechanoreceptors need to have a 4–5 h long timeframe to regain their baseline sensitivity and avoid their overstimulation/exhaustion [57,58]. This WBV treatment approach was also used in our previous experiments [50,53]. A low intensity vibration platform known as Marodyne plate (system dimensions: Height: 7.7 cm; Width: 45.7; depth: 35.6) was used (MarodyneLiv, Low Intensity Vibration; BTT Health GmbH; Germany). This system ensures a constant frequency of 30 Hz and a vertical amplitude of 50–200 μm. These parameters, along with the experimental setup, were confirmed using a 3D-accelerometer positioned at the center (frequency: 29.6 Hz; peak to peak amplitude: x: 0, y: 0, z: 0.03 mm; amplitude acceleration: x: 0.02, y: 0.01, z: 0.5 m/s^2 (or 0.001–0.05 g)) and at the corners (frequency: 29.6 Hz; peak to peak amplitude: x: 0, y: 0, z: 0.02 mm; amplitude acceleration: x: 0.01, y: 0.01, z: 0.26 m/s^2 (or 0.001–0.02 g)). The detailed experimental settings have been previously described (Oroszi et al., 2022a, 2022b). Briefly, animals (2 from the same cage) were placed in an empty cage of the same dimensions as their housing cage (36 x 18 × 23 cm)) on the top of the vibration platform. Pseudo WBV treated animals underwent the same procedure but were placed on the switched-off platform. Animals from different cages did not have social interaction during the treatments. Furthermore, animals were not habituated to the experimental settings before the intervention. Importantly, the animals did not experience any discomfort or constraint during the WBV sessions. They exhibited slight unprompted locomotor behavior during the first week. From the second week onwards, they primary remained in a sitting or lying position. Overall, we did not observe overt side effects in the rats during the WBV intervention. These observations align with our previous findings in 18 months old Wistar rats using a similar experimental approach [43,44] and they are consistent with the statement that this type of WBV protocol is safe for rodents [40].

### 2.4 Open field

**Fig. 3:**
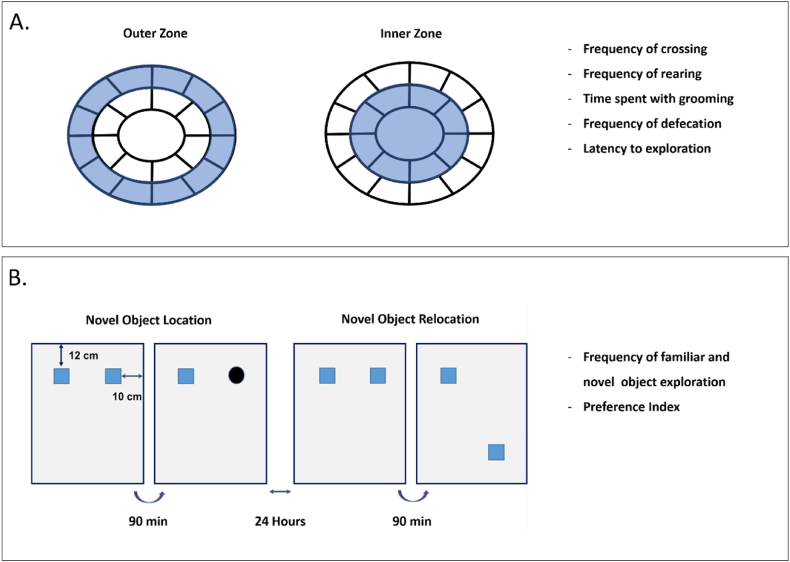
Open field (OFT) and Novel (NOR) and Spatial (SOR) Object recognition test battery. OFT was conducted to assess unprompted locomotor activity and anxiety-like behavior (Panel A). The frequency of line crossing and rearing was analyzed separately for the inner and outer zones of the area. In addition, latency to exploration, time spent with grooming and frequency of defecation were determined as secondary outcome variables. Object and spatial memory were evaluated through NOR and SOR test series (Panel B). The frequency of object exploration and preference index were determined as outcomes measures. Preference index was calculated as the proportion of exploration frequency at the novel or relocated object to total exploration frequency.

Open field test was conducted to assess anxiety-like behavior and unprompted locomotor activity induced by a novel environment [59]. The test apparatus consisted of a circular test box with a diameter of 80 cm, surrounded by a 45 cm tall aluminum wall. The test area was divided into 20 subsectors using circular and radial lines. The visual representation of the OFT area is depicted on Fig. 3 (Panel A). At the beginning of the experiment, animals were placed in the center of the area and were allowed to freely explore the test environment for a duration of 5 min. Horizontal and vertical locomotor activity were assessed by direct visual observation by an experienced researcher. The test area was cleaned by 70% ethanol and dry paper tissue following each animal's session. The following outcome measures were recorded: latency to exploration (leaving the central circle), frequency of line crossings (both in the inner and outer circles), frequency of rearing (supported and unsupported), time spent with grooming and frequency of defecation. Additionally, animals were exposed to the novel environment in a random order to minimize bias.

### 2.5 Novel and spatial object recognition

NOR and SOR tasks were performed to assess object and spatial memory [43]. The test series consisted of two separated phases (training and testing 90 min thereafter) on two consecutive days (i.e.: 24 h time gap between the two tasks). A day prior the first task, animals were habituated to the test area by being placed in the test environment for 5 min.

On the first day, animals were tested for object memory using the NOR task. Firstly, the animal was gently placed into the empty test box and habituated to the test environment for 3 min. After this habituation period, two identical objects were placed in the text box in a symmetrical position. The animal was given 5 min to freely explore these objects. This exploration period served as the training trial.

After 90 min long inter-resting interval spent in the home cage, the rat was placed back to the test area and habituated to it for 1 min. During the second session, two objects were placed back in the same symmetrical position for 5 min. However, one of the objects was replaced with a novel object. This phase served as the novel object recognition test (NOR), in which, the animal's preference for exploring the novel object over the familiar object indicates intact object memory.

The same procedure was repeated 24 h later. Animals underwent a training session, followed by a 90 min resting interval in the home cage. After the resting period, a novel object recognition test was performed. During the test, one of the two identical objects used in the training session was placed in a different position (specifically in a diagonal position) for the second session. This phase of the experiment is referred to as the spatial object recognition (SOR) task. The order of NOR/SOR tasks (i.e.: day 1 or 2) was randomly rotated throughout the experiment. Test procedure is depicted in Fig. 3 (Panel B).

Between each phase of the tasks, when the objects were removed, they were cleaned by 70% ethanol and dry paper tissue. The test box was also cleaned between each animal. Two sets of objects were used and their role was randomly assigned during the testing (i.e.: which object served as familiar or novel object). Furthermore, animals underwent both the NOR and SOR tasks in a random order to minimize potential bias. The frequency of object exploration was recorded and a preference index was calculated as the final outcome measure. Preference index was determined by using the following formula.

### 2.6 Statistical analysis

Statistica 13.2 software was used to perform statistical analysis. Student t-test was used for all parameters to reveal differences between the two experimental groups. Statistical significance was set at p ≤ 0.05. All graphs were made by GraphPad Prism 8 Software. Data are presented as mean ± SEM. Data which exceeded mean ± twice standard deviation of its group were considered as outlier (no more than 1 animal per group) and were omitted from analyses. Furthermore, the effect size was calculated by using Cohen's d factor to further examine these effects. The following benchmarks were used to interpret the effect sizes: small effect: 0.2 < d < 0.5; medium effect: 0.5 < d < 0.8; and large effect: d > 0.8.

## 3 Results

### 3.1 Open field

**Fig. 4:**
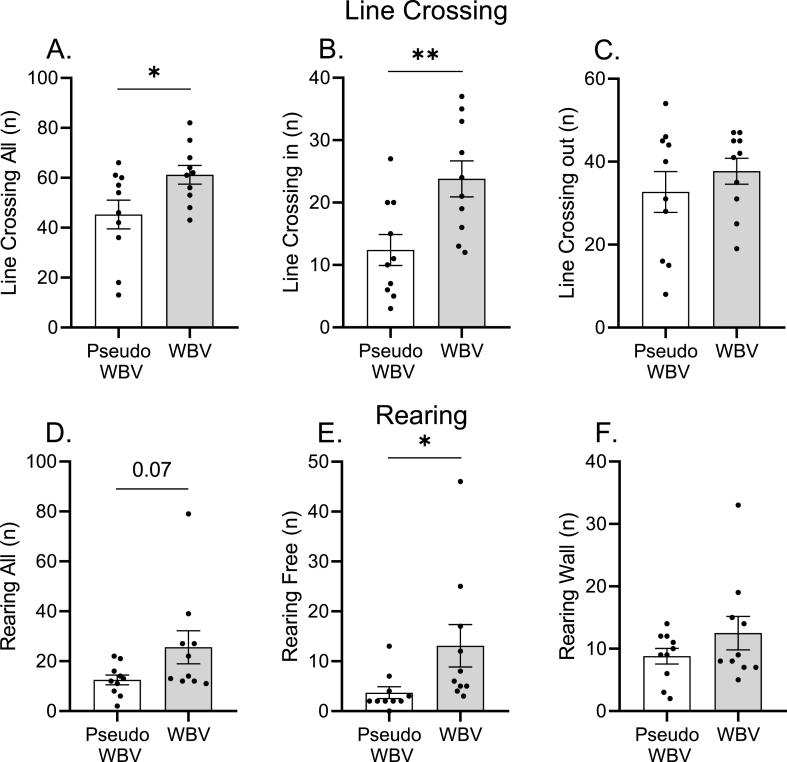
Horizontal and vertical activity in the OFT. Outcome parameters included horizontal mobility: total line crossing (Panel A), inner zone line crossing (Panel B) and outer zone line crossing (Panel C); and vertical activity: total rearing (Panel D), unsupported (panel E) and supported rearing (Panel F). Data are depicted as mean ± SEM. *p < 0.05 and **p < 0.01.

The psychomotor activity induced by the novel environment was evaluated using the OFT. Student T-test revealed a significant increase (p = 0.03) in the frequency of total line crossing in the WBV treated group compared to the pseudo WBV group (Fig. 4, Panel A). Additionally, the number of line crossings in the inner zone of the area was significantly increased by WBV treatment p ≤ 0.01) (Fig. 4, Panel B). Effect size calculation indicated that these effects were medium - large in terms of line crossing activity (total line crossing d: 0.58; line crossing in the inner zone d: 0.80). In contrast, crossing activity in the outer zone of the area did not show significant alterations (Fig. 4, Panel C).

Total number of rearing, as representing vertical activity, did not show significant change, but there was a strong tendency of increment (p = 0.07) in the WBV-treated group compared to the pseudo WBV group (Fig. 4, Panel D). However, unsupported rearing (i.e.: it refers to rearing in the non-wall zone) was significantly influenced by WBV exposure (p = 0.04) (Fig. 4, Panel E). WBV treatment significantly increased this type of rearing activity compared to the pseudo WBV group. These effects were corroborated by medium effect sizes (d > 8). In contrast, supported rearing (i.e.: it refers to rearing in the wall zone) was not significantly altered by the WBV intervention (Fig. 4, Panel F).

Secondary outcome parameters including latency to the first exploration, frequency of defecation and time spent with grooming were also significantly influenced by WBV treatment. WBV led to reduction in latency to the first exploration (p = 0.02) and frequency of defecation (p = 0.01). Moreover, WBV significantly increased the time spent with grooming (p = 0.04). These effects were supported by medium effect sizes (d > 8). The summary of these secondary outcome parameters can be found in Table 1.

**Table 1:** Secondary outcomes of the OFT: latency to start the exploration, time spent with grooming and frequency of defecation. Latency of exploration and frequency of defecation was significantly decreased after WBV. In contrast, time spent with grooming was significantly increased in the WBV treated group.

Behavior	Intervention	Mean +SEM	P Value	WBV Effect
Latency to exploration	WBV	4.20 ± 2.27	0.02	Reduced Anxiety
	Pseudo WBV	9.80 ± 0.53		
Grooming	WBV	41.20 ± 4.62	0.04	More relaxed
	Pseudo WBV	28.30 ± 3.81		
Frequency of defecation	WBV	2.60 ± 0.58	0.01	Decreased Fear
	Pseudo WBV	5.38 ± 0.83		

### 3.2 Novel and spatial object recognition

**Fig. 5:**
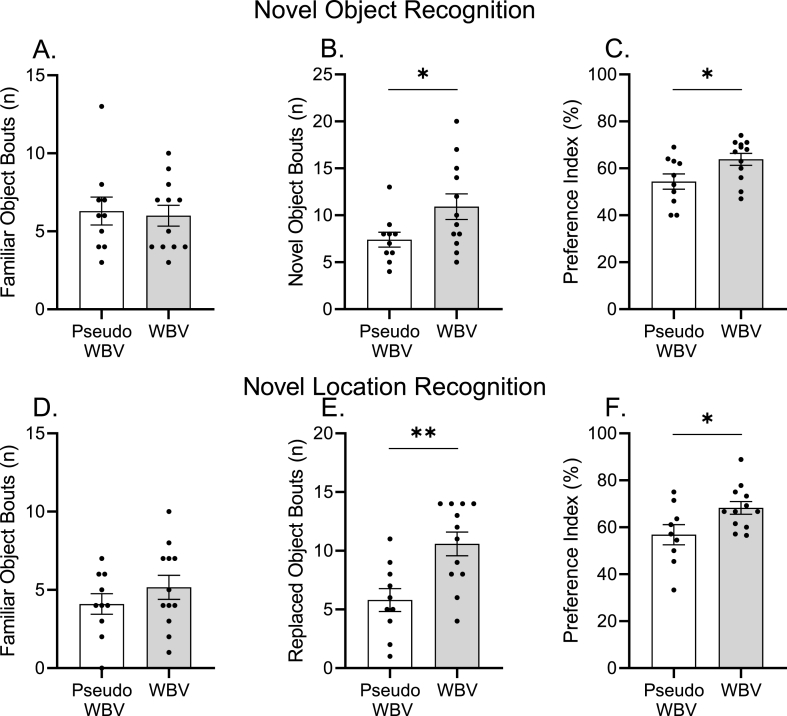
Novel and spatial object memory performance in novel object (NOR) and location (SOR) recognition tests. Outcome parameters were determined as NOR: frequency of familiar (Panel A) and novel (Panel B) object exploration, and preference index (Panel C); and SOR: frequency of familiar (Panel D) and replaced (Panel E) object exploration and preference index (Panel F). Data are depicted as mean ± SEM. *p < 0.05 and **p < 0.01.

We used a battery of NOR and SOR tasks to characterize alterations in object and spatial memory functions. In the NOR task, the WBV treated animals exhibited a significantly higher number of bouts of exploration of the novel object compared to the pseudo WBV group (p = 0.04) (Fig. 5, panel B). In contrast, there was no difference for the familiar object (Fig. 5, panel A). This improvement was also found by the novel object preference index, which was significantly higher in the WBV treated group (p = 0.03) (Fig. 5, panel C).

Similarly, in the SOR task, the WBV treated group showed a significant increase in the exploration frequency (number of bouts) for the replaced object compared to the pseudo WBV group (p = 0.003), while no difference was observed for the familiar object (Fig. 5, panel D and E). The preference index in the SOR task was also significantly increased in the WBV treated group (p = 0.02) (Fig. 5, panel F). Notably, no difference was observed in the frequency of exploration or preference index during the training phases. The effects observed in both NOR and SOR tasks were corroborated by medium – large effect sizes (5 < d > 9). additionally, the body weight of the animals remained unchanged during the intervention.

## 4 Discussion

The present experiment examined the effects of long-term (5 weeks) WBV intervention with low intensity of a sinusoidal nature on memory functions and anxiety-like behavior in senescent rats. The main findings of this study indicate that WBV is an effective intervention to induce behavioral responses in 30 months old senescent rats such as increased exploration, reduced fear and anxiety-like behavior in the OFT and improved memory functions in the NOR and SOR tasks. These data indicate that both spontaneous activity in a novel environment and cognitive capabilities in different memory tasks can be ameliorated by WBV. As such, our data demonstrated that 5 min WBV exposure twice daily for 5 consecutive weeks using the MarodyneLiv vibration system is a viable strategy to improve age-related increase of anxiety-like behavior and decline of memory functions.

Significant improvements were observed in both horizontal and vertical activity, as indicated by an increased frequency of line crossing and unsupported rearing during the OFT. Additionally, WBV intervention resulted in attenuated emotionality as evidence by a decrease in freezing behavior and defecation rate, and an increase in grooming behavior. It is worth mentioning that these outcome measures in the OFT are age-dependent [[17], [18], [19]]. Specifically, rats over 30 months of age typically exhibit an exponential decline in rearing and walking activities compared to younger age groups (20–25 months) [18]. It might be assumed that this particular deterioration in the motor regulation of the hind limbs is accompanied by increased muscular atrophy and insufficient muscle strength. Furthermore, previous studies have shown that unloading the hind limbs in rats leads to progressive muscle mass loss and a concomitant increase in protein degradation [60]. However, the increased unsupported rearing (i.e.: rearing in the non-wall zone) observed in the WBV-treated group may be indicate not only of an improved motor performance, but also of reduced stress and anxiety, as unsupported rearing has been associated with increased time spent in the center and sensitivity to acute stress. This previous finding may add to the understanding why WBV was more effective in case of unsupported rearing. Other outcome parameters of the OFT such as defecation rate, grooming and freezing behavior are indicators of the animal's emotional states, particularly fear [19]. In the present study, we may assume that WBV treatment shares similar benefits in both movement coordination and muscle strength in the hind limbs, as well as in anxiety-like behavior, as evidence by the overall improvements in the OFT outcomes. This reflects the well-known combined effects of WBV therapy on brain and motor functions [[36], [37], [38]]. Off note, it is worth mentioning that WBV therapy has previously been shown to enhance muscle strength and coordination in various involuntary motor task in rats [43,44].

To the best of authors’ knowledge, there have been six published studies investigating the effects of WBV on anxiety-like and exploratory behavior in the OFT using rats [41,[43], [44], [45],^53^,^61^]. In two of our previous studies, we conducted long-term (5 weeks) WBV interventions with similar experimental designs, involving the Marodayne plate for daily sessions of 5–10- or 20-min over the time course of 5 consecutive weeks [43,44]. These interventions successfully improved anxiety-like behavior in 18 months old Wistar rats. Specifically, five-week intervention with low intensity (frequency of 30 Hz, amplitude of 50–200 μm) was successfully applied to increase rearing activity, while line crossing activity remained unaffected in these experiments [43,44]. In another study conducted by our research group related to post operative cognitive decline, a two-week WBV intervention with the same intensity did not influence rearing and line crossing activities compared to the surgery – control animals [53]. In addition, we recently reported the lack of effectiveness of a 5-weeks long WBV intervention following isoproterenol-induced cardiac infarction in young Wistar rats [61]. Nevertheless, our current finding supports the effectiveness of WBV interventions in the context of age-related decline, which corraborates with our previous observation suggesting that WBV might become a more effective treatment strategy when cognitive and motor functions are significantly deteriorated. In addition, similar therapeutic effects of long-term WBV interventions on anxiety-like behavior induced by restraint stress model or middle cerebral artery occlusion have been demonstrated by other researchers [41,45]. It is important to note that these studies have used different vibration parameters such as frequencies of 30 and 40 Hz and amplitude of 4.5 mm (amplitude is not reported in Kerr et al.). When compared to our own protocol (frequency of 30 Hz and amplitude of 50–200 μm), this finding seems to confirm our previous observation that the proper adjustment of frequency (of 30–40 Hz) may play a more crucial role to obtain benefits on anxiety-like behavior in rats compared to amplitude [43,44].

In the NOR and SOR tasks, both novel object and spatial object memory increased as seen in the preference index, and also the exploration frequency of novel or relocated objects were significantly improved by WBV treatment. Ample studies have demonstrated that rodents have age-related deficits in their learning and memory capabilities [[13], [14], [15], [16]]. We previously reported that 18 months old rats subjected to 5 weeks of WBV were able to memorize a relocated object in the SOR task, but not a novel object in the NOR task [43,44]. This observation may be attributed by the altered sensitivity of spatial and object memory functions to the aging process. Advanced aging is known to impair both spatial and object memory functions, although the exact time course of these age-related declines is still far from being well characterized. It is assumed that impairments in spatial object memory become detectable as early as 15 months of age, while object memory seems to be preserved until 15 months of age but declines gradually thereafter [62]. Similar observations have been presented by other researchers as well [63,64]. Overall, our findings seem to be in line with these observations and indicate the beneficial effects of WBV on spatial and object memory functions during progressive aging. Other studies demonstrated that WBV therapy works in a similar way, by mitigating the decline in spatial memory in rats induced by chronic restraint test [41] and cerebral artery occlusion [45]. Furthermore, similar to our findings in the OFT, WBV did not alter either spatial or object memory after major abdominal surgery [53] or isoproterenol-induced cardiac infarction [61].

It is important to note that translational research utilizing rats as experimental subjects represents an important approach to study and understand (underlying) brain mechanisms. The relevance of this approach has been emphasized by multiple reviews [36,37,39]. However, it is important to acknowledge that the number of studies in this field is still relatively limited. To this day, with only approximately ∼170 studies have been done with rats as experimental subjects. Among these studies, ∼40 have been specifically focused on brain functions. Furthermore, it should be mentioned that 8–10 studies have been published with similar methodical approaches including OFT and NOR/SOR tasks. Consequently, the available literature in this context remains limited.

Taking together, our findings suggest that WBV can ameliorate age-related decline in both spatial and object memory. These beneficial effects induced by WBV may vary depending on the age of the subjects. Although our study primarily focused on the behavioral aspects of WBV research, our results indirectly support the theory that WBV exposure can stimulate the hippocampus and the prefrontal cortex (PFC). It is well known that spatial memory and navigation are strongly associated with hippocampal functioning [65], while consolidation of object memory mainly relies on the functional and structural integrity of the PFC [66]. Previous literature has shown that WBV can have beneficial effects on the hippocampus in rats, including mitigation of pathological alterations in glial cells, upregulation of neurotrophic factors and enhancement of synaptic plasticity [41,[44], [45], [46],^49^]. Similar effects on hippocampal functioning have also been observed in mice [42,51,[67], [68], [69]]. In contrast, the effects of WBV on PFC functioning are even less explored and require further investigation.

Lastly, it should be noted that various forms of active exercise interventions have been extensively demonstrated to prevent age-dependent decline in memory functions, anxiety-like and exploratory behavior in aged rodents [28,29,70,71]. Hence, WBV appears to be a promising alternative that can partly replicate beneficial effects of active exercise interventions. Taken together, there is good agreement among the behavioral data collected from this and our previous studies, and those obtained from other researchers, further supporting the effectiveness of WBV as a potential therapeutic approach.

## 5 Limitations

Several limitations need to be addressed. Firstly, the present study only included rats at the age of 30 months, and can be directly compared to our previous research with WBV in 18 months old Wistar rats [43,44] However, considering the interaction between aging and therapeutic potential of WBV as form of passive exercise as our main hypothesis, involving different age groups at the same time to dive into the progression of aging could be considered to further investigate the progression of aging. We also would like to mention that using 32 months old rats can be challenging per se as it takes 2.5 years of maintaining the animals in the facility before the experiment can start.

Secondly, conducting baseline behavioral testing prior the start of the intervention could have provided valuable insights into the baseline levels of anxiety and cognitive functions and their age-related progression. However, it is important to note that it is well-documented that repeated testing procedures may introduce some discrepancies in the results.

Finally, it is crucial to highlight that the field of WBV research is still expanding and preclinical data focusing on the CNS and its related functions in rats are limited. Therefore, follow-up experiments are needed to further enhance the interpretation of these findings and to reveal the underlying mechanisms in more detail.

## 6 Conclusion

This study aimed to investigate the potential translational value of chronic low-intensity WBV intervention in rats reaching the senescent age of 30 months. The primary aim was to explore the effects of WBV on the concomitant age-related increase in anxiety-like behavior and decline in cognitive functions. The findings demonstrated that WBV effectively attenuated anxiety-like behavior and improved unprompted locomotor activity in the OFT. Moreover, WBV treatment also enhanced cognitive functions related to object and spatial recognition, indicating its efficacy in memory tasks.

In accordance with our previous studies, our current findings also indicate the effectiveness of WBV treatment in improving anxiety-like indices and cognitive abilities in senescent rats. It contributes to the growing literature of emphasizing WBV as an intervention that has the potential to stimulate the brain. Furthermore, future translational studies need to pay attention to reveal and understand the underlying molecular and cellular processes related to brain functioning.

## Funding

This work was supported by the Higher Education Institutional Excellence Program at 10.13039/501100002332Semmelweis University, Hungary.

## Data statement

All data generated in this experiment are available in its supplementary material.

## CRediT authorship contribution statement

**Tamás Oroszi:** Writing – original draft, Visualization, Formal analysis, Data curation. **Klára Felszeghy:** Writing – review & editing, Data curation. **Paul G.M. Luiten:** Writing – review & editing, Conceptualization. **Regien G. Schoemaker:** Writing – review & editing, Conceptualization. **Eddy A. van der Zee:** Writing – review & editing, Supervision, Funding acquisition, Conceptualization. **Csaba Nyakas:** Writing – review & editing, Supervision, Funding acquisition, Conceptualization.

## Declaration of competing interest

The authors declare that they have no known competing financial interests or personal relationships that could have appeared to influence the work reported in this paper.
