# Supplementary material for: Whole body vibration ameliorates anxiety-like behavior and memory functions in 30 months old senescent male rats
Source: Heliyon. 2024 Feb 16;10(4):e26608. doi: 10.1016/j.heliyon.2024.e26608 (PMC10884920; doi:10.1016/j.heliyon.2024.e26608)
Supplement: Multimedia component 1 [file mmc1.docx]

|  |  |  | **N** | **Mean** | **Median** | **Min** | **Max** | **SD** | **SEM** |
| --- | --- | --- | --- | --- | --- | --- | --- | --- | --- |
| **Open**  **Field** | **Lat - Sec** | Control | 10 | 9.800000 | 7.000000 | 4.000000 | 28.00000 | 7.208020 | 2.279376 |
|  |  | WBV | 10 | 4.200000 | 3.500000 | 3.000000 | 8.000000 | 1.686548 | 0.533333 |
|  | **Crossing**  **Out** | Control | 10 | 32.70000 | 35.50000 | 8.000000 | 54.00000 | 15.59950 | 4.932995 |
|  |  | WBV | 10 | 37.70000 | 41.50000 | 19.00000 | 47.00000 | 9.843780 | 3.112876 |
|  | **Crossing**  **In** | Control | 10 | 12.40000 | 10.50000 | 3.000000 | 27.00000 | 7.862711 | 2.486407 |
|  |  | WBV | 10 | 23.80000 | 22.50000 | 12.00000 | 37.00000 | 9.126275 | 2.885981 |
|  | **Crossing**  **All** | Control | 10 | 45.30000 | 50.00000 | 13.00000 | 66.00000 | 18.22727 | 5.763969 |
|  |  | WBV | 10 | 61.20000 | 61.50000 | 43.00000 | 82.00000 | 11.91451 | 3.767699 |
|  | **Rearing**  **Wall** | Control | 10 | 8.800000 | 9.500000 | 2.000000 | 14.00000 | 3.966527 | 1.254326 |
|  |  | WBV | 10 | 12.50000 | 8.500000 | 5.000000 | 33.00000 | 8.436034 | 2.667708 |
|  | **Rearing**  **Free** | Control | 10 | 3.700000 | 2.000000 | 0.00 | 13.00000 | 3.743142 | 1.183685 |
|  |  | WBV | 10 | 13.10000 | 7.000000 | 3.000000 | 46.00000 | 13.46972 | 4.259499 |
|  | **Rearing**  **All** | Control | 10 | 12.50000 | 12.50000 | 2.000000 | 22.00000 | 6.258328 | 1.979057 |
|  |  | WBV | 10 | 25.60000 | 18.00000 | 11.00000 | 79.00000 | 20.88168 | 6.603366 |
|  | **Grooming** | Control | 10 | 28.30000 | 26.50000 | 11.00000 | 48.00000 | 12.06510 | 3.815320 |
|  |  | WBV | 10 | 41.20000 | 40.00000 | 18.00000 | 65.00000 | 14.63481 | 4.627935 |
|  | **Bolus** | Control | 10 | 5.380000 | 5.000000 | 2.000000 | 9.800000 | 2.637254 | 0.833973 |
|  |  | WBV | 10 | 2.600000 | 2.000000 | 0.00 | 5.000000 | 1.837873 | 0.581187 |
| **NOR**  **Training** | **Left Total** | Control | 10 | 7.200000 | 6.000000 | 3.000000 | 15.00000 | 3.583915 | 1.133333 |
|  |  | WBV | 12 | 8.083333 | 7.500000 | 4.000000 | 16.00000 | 3.604501 | 1.040530 |
|  | **Right Total** | Control | 10 | 6.500000 | 6.500000 | 3.000000 | 11.00000 | 2.838231 | 0.897527 |
|  |  | WBV | 12 | 8.500000 | 8.000000 | 5.000000 | 13.00000 | 2.354879 | 0.679795 |
|  | **Preference** | Control | 10 | 48.30000 | 49.00000 | 40.00000 | 57.00000 | 6.165315 | 1.949644 |
|  |  | WBV | 12 | 52.57500 | 54.00000 | 38.00000 | 69.00000 | 9.604840 | 2.772679 |
| **NOR**  **Test** | **Familiar** | Control | 10 | 6.300000 | 6.000000 | 3.000000 | 13.00000 | 2.830391 | 0.895048 |
|  |  | WBV | 12 | 6.000000 | 6.000000 | 3.000000 | 10.00000 | 2.296242 | 0.662868 |
|  | **Novel** | Control | 10 | 7.400000 | 7.500000 | 4.000000 | 13.00000 | 2.503331 | 0.791623 |
|  |  | WBV | 12 | 10.91667 | 9.500000 | 5.000000 | 20.00000 | 4.699291 | 1.356568 |
|  | **Preference** | Control | 10 | 54.40000 | 55.00000 | 40.00000 | 69.00000 | 10.27619 | 3.249615 |
|  |  | WBV | 12 | 63.83333 | 67.50000 | 47.00000 | 74.00000 | 8.788767 | 2.537098 |
| **NOR** | **Bolus** | Control | 10 | 5.000000 | 5.500000 | 0.00 | 12.00000 | 3.800585 | 1.201850 |
|  |  | WBV | 12 | 1.916667 | 3.000000 | 0.00 | 4.000000 | 1.729862 | 0.499368 |
| **NLR**  **Training** | **Left Total** | Control | 10 | 5.600000 | 4.500000 | 1.000000 | 14.00000 | 3.977716 | 1.257864 |
|  |  | WBV | 12 | 4.416667 | 3.500000 | 0.00 | 11.00000 | 3.502164 | 1.010988 |
|  | **Right Total** | Control | 10 | 5.000000 | 5.000000 | 3.000000 | 8.000000 | 1.333333 | 0.421637 |
|  |  | WBV | 12 | 5.750000 | 4.500000 | 2.000000 | 12.00000 | 3.414541 | 0.985693 |
|  | **Preference** | Control | 10 | 52.76679 | 50.00000 | 33.33333 | 80.00000 | 15.40891 | 4.872725 |
|  |  | WBV | 12 | 56.20058 | 56.34921 | 37.50000 | 71.42857 | 10.73708 | 3.099529 |
| **NLR**  **Test** | **Familiar** | Control | 10 | 4.100000 | 4.000000 | 0.00 | 7.000000 | 2.078995 | 0.657436 |
|  |  | WBV | 12 | 5.166667 | 4.500000 | 1.000000 | 10.00000 | 2.657180 | 0.767062 |
|  | **Replaced** | Control | 10 | 5.800000 | 5.500000 | 1.000000 | 11.00000 | 3.084009 | 0.975249 |
|  |  | WBV | 12 | 10.58333 | 11.50000 | 4.000000 | 14.00000 | 3.502164 | 1.010988 |
|  | **Preference** | Control | 9 | 56.85025 | 57.14286 | 33.33333 | 75.00000 | 12.94883 | 4.316278 |
|  |  | WBV | 12 | 68.28615 | 66.66667 | 56.52174 | 88.88889 | 9.393615 | 2.711703 |
| **NLR** | **Bolus** | Control | 10 | 2.700000 | 2.000000 | 0.00 | 8.000000 | 2.668749 | 0.843933 |
|  |  | WBV | 12 | 4.500000 | 5.000000 | 0.00 | 9.000000 | 3.605551 | 1.040833 |

|  |  | **T-value** | **df** | **p** | **Observed Power** |
| --- | --- | --- | --- | --- | --- |
| **Open Field** | **Lat - Sec** | -2.39220 | 18 | 0.027869 | 0.619 |
|  | **Crossing**  **Out** | 0.857185 | 18 | 0.402612 | 0.128 |
|  | **Crossing**  **In** | 2.992640 | 18 | 0.007809 | 0.807 |
|  | **Crossing**  **All** | 2.308987 | 18 | 0.033022 | 0.588 |
|  | **Rearing**  **Wall** | 1.255139 | 18 | 0.225480 | 0.221 |
|  | **Rearing**  **Free** | 2.126259 | 18 | 0.047573 | 0.520 |
|  | **Rearing**  **All** | 1.900326 | 18 | 0.073529 | 0.436 |
|  | **Grooming** | 2.150764 | 18 | 0.045328 | 0.530 |
|  | **Bolus** | -2.73485 | 18 | 0.013605 | 0.734 |
| **NOR**  **Training** | **Left** | 0.573819 | 20 | 0.572488 | 0.084 |
|  | **Right** | 1.807938 | 20 | 0.085680 | 0.405 |
|  | **Preference** | 1.212158 | 20 | 0.239578 | 0.211 |
| **NOR**  **Test** | **Familiar** | -0.27471 | 20 | 0.786356 | 0.057 |
|  | **Novel** | 2.123049 | 20 | 0.046422 | 0.524 |
|  | **Preference** | 2.322283 | 20 | 0.030885 | 0.598 |
| **NOR** | **Bolus** | -2.52309 | 20 | 0.020211 | 0.670 |
| **SOR**  **Training** | **Left** | -0.74218 | 20 | 0.466602 | 0.108 |
|  | **Right** | 0.652225 | 20 | 0.521682 | 0.095 |
|  | **Preference** | 0.614620 | 20 | 0.545735 | 0.090 |
| **SOR**  **Test** | **Familiar** | 1.031896 | 20 | 0.314439 | 0.165 |
|  | **Novel** | 3.364374 | 20 | 0.003084 | 0.892 |
|  | **Preference** | 2.351007 | 19 | 0.029670 | 0.606 |
| **SOR** | **Bolus** | 1.306402 | 20 | 0.206241 | 0.237 |
